# Nonsynonymous Synonymous Variants Demand for a Paradigm Shift in Genetics

**DOI:** 10.2174/1389202924666230417101020

**Published:** 2023-06-23

**Authors:** Mauno Vihinen

**Affiliations:** 1 Department of Experimental Medical Science, Lund University, Lund, BMC B13, Sweden

**Keywords:** Synonymous variation, silent variation, unsense variation, phylogenetics, distribution of fitness effects, nonsynonymous-to-synonymous substitution ratio

## Abstract

Synonymous (also known as silent) variations are by definition not considered to change the coded protein. Still many variations in this category affect either protein abundance or properties. As this situation is confusing, we have recently introduced systematics for synonymous variations and those that may on the surface look like synonymous, but these may affect the coded protein in various ways. A new category, unsense variation, was introduced to describe variants that do not introduce a stop codon into the variation site, but which lead to different types of changes in the coded protein. Many of these variations lead to mRNA degradation and missing protein. Here, consequences of the systematics are discussed from the perspectives of variation annotation and interpretation, evolutionary calculations, nonsynonymous-to-synonymous substitution rates, phylogenetics and other evolutionary inferences that are based on the principle of (nearly) neutral synonymous variations. It may be necessary to reassess published results. Further, databases for synonymous variations and prediction methods for such variations should consider unsense variations. Thus, there is a need to evaluate and reflect principles of numerous aspects in genetics, ranging from variation naming and classification to evolutionary calculations.

## 1 INTRODUCTION

Nucleotide substitutions in RNA have traditionally been grouped into three categories: synonymous, missense, and nonsense variations; however, there is a need for a fourth category. Synonymous (also called silent) variations are defined as those that do not change the coded amino acid, while missense variants have a nucleotide difference that causes an amino acid substitution in the coded protein. Nonsense variant introduces a premature stop codon, which when early leads usually to mRNA degradation by quality control mechanisms, typically by nonsense-mediated decay (NMD) [1, 2]. It is quite common and wrong to use terms like nonsense and missense to describe changes in DNA or protein level [3, 4].

Many variants annotated as synonymous are not synonymous at all [5-9]. The language for synonymous variants is often confusing and misleading. Therefore, we have recently presented systematic for synonymous variations and introduced a new category of unsense variation [7]. Unsense variation is a substitution in the mRNA coding region that affects gene expression, protein, or protein production without introducing a stop codon in the variation site. These variants are not synonymous or silent and indeed have an effect on the coded protein. The definition has been implemented in Variation Ontology (VariO), which is used for the systematic description of effects, types, consequences, and mechanisms of biological variations [10]. When focusing on the genetic code, a variant may seem synonymous, but it may still affect the protein; thus, calling such variants as synonymous is incorrect.

In the following paper, notation “synonymous” variation is used to indicate cases where unsense variants have not been separated from synonymous variants.

In this article, the consequences of including unsense variants in various types of studies are discussed. This exerts an important effect on variation interpretation and genetic disease diagnosis. These variants are typically misannotated [11]. In addition, unsense variants have to be included in evolutionary inference and in phylogenetic and natural selection predictions. Since synonymous variants are typically considered as neutral or nearly neutral, it is necessary to re-evaluate and sometimes to re-analyze studies based on this assumption. Some authors claim synonymous variants to have a small effect [12], while there is a lot of compelling evidence that many synonymous variants affect the coded protein and have functional and other effects [8, 13, 14]. Databases for “synonymous” variants and predictors for such variations mix different types of variations [15-18]. Thus, a paradigm shift is needed in many genetic studies and approaches.

## 2 UNSENSE AND SYNONYMOUS VARIATIONS

**Fig. (1):**
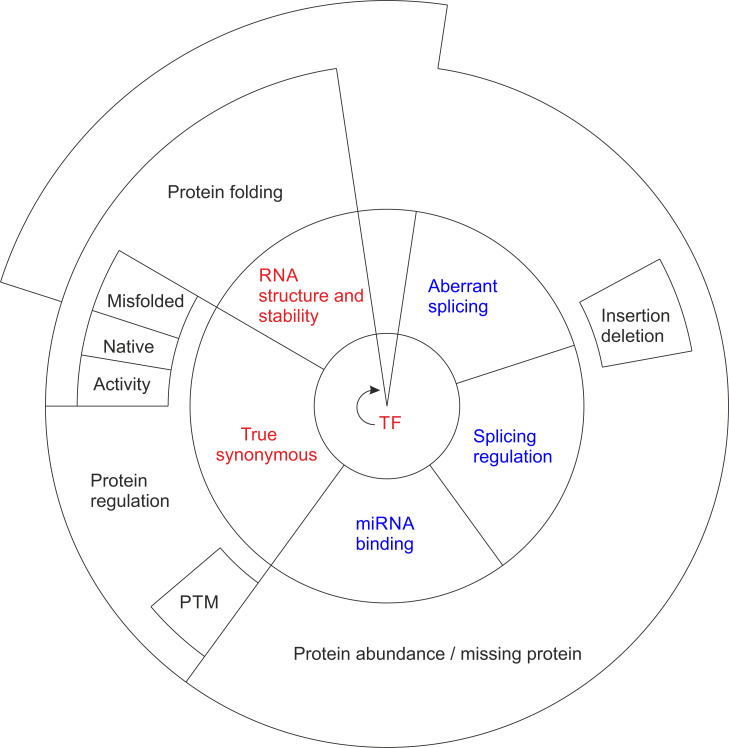
Classification of synonymous (red) and unsense (blue) variants and their consequences at DNA (center), RNA (middle), and protein (outer circle) levels. The inner circle indicates DNA variants, which although synonymous can affect transcription factor (TF) binding, and thereby protein production. The middle circle depicts mRNA level alterations, and on the outer ring are shown the protein level effects. At mRNA, synonymous variants are either true synonymous or those that affect RNA structure and stability. Synonymous variants at the protein level have either native or misfolded structure or affect protein activity. Changes to protein abundance are common. PTM indicates post-translational protein modification. The three categories of unsense variants (blue) affect aberrant splicing, splicing regulation, or miRNA binding. Many of these variants have an effect on protein abundance, and often there is no protein at all. Aberrant splicing and splicing regulation affecting variants can lead also to protein insertion or deletion, if the variant position is close to the C-terminus or the mRNA is not degraded by nonsense-mediated decay.

Systematics has been presented for synonymous and unsense variants [7]. These variants can have effects on DNA, RNA, and/or protein level (Fig. **1**). On the DNA level, synonymous variants can affect transcription factor binding and consequently gene expression, without altering the protein sequence.

On the mRNA level, the synonymous and synonymous-like variants can be divided into three major categories (Fig. **1**). True synonymous variations are the category that this group of variations is traditionally considered to describe. Although these variants do not affect coded protein sequence, there are variants that have effects on protein regulation, post-translational modifications, protein structure or activity (Fig. **1**). The second group is classified as synonymous, but they affect RNA structure and stability. These variants affect the folding and abundance of the coded protein, as shown in the studies presented earlier [5-7, 19].

The third category includes unsense variants. Three types of unsense variants are known. They affect either splicing, splicing regulation, or miRNA binding due to exon variations [7]. Unsense variants are important and apparently cover a substantial portion of variations, also among those causing diseases. Based on the universal codon table, 23.8% of possible substitutions are for the same amino acid-coding codons [7]. The situation is somewhat different in genes due to different vulnerability of nucleotides, nucleotide composition, gene C+G content, *etc*. [20]. The consequences of variations, whether synonymous or unsense, depend on many factors, and the context of the variants also plays an important role [7].

Currently, three mechanisms behind unsense variants are known [7], but there may be more. Splicing-affecting unsense variants are not synonymous due to aberrant mRNA splicing; they often lead to frameshift alterations, are recognized by NMD machinery, and are degraded. Therefore, the variant is not synonymous and no protein is produced. Those mRNAs that are not degraded code for altered protein due to aberrant splicing [3]. Unsense variants inactivate exonic splice sites or activate cryptic splice sites [21], alter exonic splice site regulators (exonic splice site enhancers (ESEs) [22] or exonic splicing silencers (ESSs) [23]), or modify regulatory exonic miRNA binding sites [24-26].

How frequent are unsense variants? It is not possible to give an exact estimate as it depends on many factors, being different for different genes; however, examples are available in the literature. Of all the possible synonymous variants in exon 7 in the *SMN1* gene, 32 out of 138 variants (23%) decrease exon inclusion [8]. An analysis of 66 out of 67 possible synonymous variations in exon 6 of the *TP53* gene for TP53 protein indicated that nine (13%) variants had a large decrease in splicing [27] due to exon skipping, intron inclusion, or exon truncation. A total of 6.3% of 725 *de novo* coding region variants, which have been identified in autism spectrum disorder families, disrupted splicing [13] and included “synonymous” variants

Recently, Shen and coworkers presented an interesting, systematic study of the fitness effects of thousands of single nucleotide variants on 21 *Saccharomyces cerevisiae* genes [14]. They showed that the majority of synonymous variants had a strong fitness effect, and many of them had an effect on gene expression. In conclusion, we can say that variants that have been classified as synonymous, but which in reality are not synonymous, are frequent, and they often affect splicing and protein abundance. Thus, there is a need for the new classification of variants claimed to be synonymous and for the new term unsense variant (Fig. **1**).

## 3 PROBLEMS WITH MISCLASSIFICATION OF “SYNONYMOUS” VARIATIONS

Due to a lack of awareness of unsense variants, they are incorrectly annotated, for *e.g*., in sequencing projects [11]. They are ignored and lumped together with true synonymous variants by variation annotation tools. For example, ANNOVAR [28], SnpEff [29], and Variant Effect Predictor (VEP) [30] have just one category for synonymous/silent variants. In variation interpretation, these variants are usually ignored, and therefore, disease diagnosis may be prevented or substantially delayed, which may have severe consequences for the patients.

One of the problems with “synonymous variations” was indicated in the title of the News and Views piece describing the work of Shen and others [14]: “Mutations matter even if proteins stay the same” [31]. In the case of “synonymous” variants, the proteins do not always stay the same, and there may not be any protein at all.

## 4 FITNESS EFFECTS OF “SYNONYMOUS” VARIATIONS

Fitness effects of variations, including ”synonymous” variants, have been investigated experimentally in several organisms and genes [9]. These studies have been conducted in viruses, bacteria, and fungi, and widely indicate the variable distribution of fitness effects (DFEs). The DFE scores of synonymous variants can be the same or even lower than those for non-synonymous variants. Thus, many “synonymous” variants are likely not synonymous. The mechanisms are unknown; splicing-related unsense variants do not occur as viruses and bacteria do not contain introns and have splicing. In the case of yeast, at least some of these observations are likely due to unsense variants.

We argue that in the extensive study of 21 yeast genes [14], a substantial number of the “synonymous” variants that have non-neutral fitness effects are in fact unsense variants. Shen and colleagues investigated, among others, 1866 “synonymous” variants, which showed fitness effects quite similar to missense variants. According to the neutral theory of synonymous variants, these observations cannot be interpreted. To elucidate mechanisms for the effects, relative expression levels (RELs) of variant proteins need to be investigated.

The RELs of altogether 53.8% of the “synonymous variants” deviated significantly from 1 [14], the score for normal gene expression. It is likely that the majority of these instances are not synonymous at all, but affect splicing or regulation, and are thus unsense variants. It would be interesting to sequence the mRNAs to study splicing aberrations for those variants that have residual mRNA. As the performances of prediction methods for consequences of exonic variants beyond the immediate exon-intron boundary are rather poor, these methods would likely not be applicable here. Therefore, a pragmatic way to investigate the data of Shen *et al.* [14] would be to classify the “synonymous” variants with significant REL deviation from 1 as unsense variants and repeat the analyses for the four variant classes.

The fact that more than 50% of “synonymous” variants can behave against the assumption of the neutral theory of synonymous variants indicates that the variant naming is not correct and a new classification is needed. Further, various predictions based on the assumption have to be re-assessed as the foundations do not hold.

## 5 NONSYNONYMOUS TO SYNONYMOUS SUBSTITUTION RATIO

One area based on the assumption that synonymous variants are (nearly) neutral is a calculation of nonsynonymous-to-synonymous substitutions ratios [32] (marked as *ω*, *dN/dS* or *Ka/Ks*). This score has been used as the most common measure of the strength and the mode of natural selection of genes. Several algorithms with codon models and additional properties and assumptions have been implemented [33, 34]. These widely used scores are calculated from multiple sequence alignments of related sequences and are prone to confounding effects, for *e.g*., because of the choice of sequences, their similarities/identities, codon frequencies, how different nucleotide models are handled, *etc*.

Problems with “synonymous” variations in these scores have been known and discussed [35] and remedies have been suggested. However, the actual reason, the heterogeneity of “synonymous” variations, has not been fully considered. It is now evident that these kinds of calculations include unsense variants and have to apply more complex and more realistic models. Therefore, it is necessary to evaluate and, when necessary, reassess published predictions of codon substitution model-based estimates of natural selection.

## 6 PHYLOGENETICS AND EVOLUTIONARY MODELING

Substitution models are used in evolutionary biology to describe alterations during time, *i.e*., rate of change of variations. These models are at the core of phylogenetic inference and other evolutionary biology applications, including calculations of loss and gain of genes (gene turnover) [36] and nonsynonymous-to-synonymous substitution ratios. Substitution models can be applied to nucleotide or amino acid sequence data. General time reversible (GTR) family of nested models is widely used in maximum likelihood algorithms [33]. In addition to individual rates for the variations and nucleotide frequencies, additional details of invariable sites, variation across sites, neighbor interactions, *etc*., are used. Model selection is a critical step in evolutionary inference.

As unsense variations have not been included in the substitution models, it is necessary to evaluate their contribution to the models as well as generate predictions, such as phylogenies. “Synonymous” variations account for theoretically over 20% of single nucleotide substitutions, and as a substantial portion of these is unsense cases, they are an important variant category.

## 7 DATABASES AND PREDICTORS

Unsense variants are misclassified in databases for “synonymous” variants. For example, the Database of Deleterious Synonymous Mutations (dbDSM) [18] mainly contains unsense variants, not synonymous ones. This database has also another problem. It contains a large number of markers used in genome-wide association studies (GWASs). Even if the markers are synonymous, it is not relevant for the property as the markers, or tags, hardly ever are related to the associated property, they are just markers for the haplotypes that contain the associated variation.

Regarding cancers, two resources contain massive amounts of “synonymous” variation information, SynMicDb [37] and DMSN [17]. Even these resources do not differ between unsense and synonymous variants. The availability of these data facilitates further studies of some cases. Several prediction methods have been released for synonymous variants; however, the cases used for training and developing these tools are mainly unsense variants.

Several methods claim to predict the outcome of synonymous variants, including DDIG-IN [38], EnDSM [39], IDSV, an ensemble approach [40], prDSM [41], Silva [42], Syntool [43], regSNPs-splicing [44], and Transcipt-inferred Pathogenicity (TraP) [45]. The cases used to train and develop these methods are mainly for unsense variants and affect splicing.

Tools dedicated to true synonymous variants are missing and those trained with unsense variants are not optimal for these cases as the effects are not considered, and variant distribution is biased.

## CONCLUSION

The traditional category of “synonymous” variants also contains unsense variants; therefore, it is necessary to re-evaluate the relevance of the results of some prior studies. In forthcoming investigations, unsense variants should be included.

The introduction of the concept of unsense variants facilitates an understanding of systematic annotation and requires changes in annotation tools [28, 29] and variation interpretation [46]. Existing studies that rely on the neutrality in various codon indices, as well as those on phylogenetic inference and evolutionary modelling need to be reassessed. Although information and examples of non-neutral synonymous variants have been around for years, new methods are needed with more realistic assumptions and premises, including unsense variants. Therefore, it is necessary to check the foundations of these studies and include unsense variations in the models, programs, and algorithms. This is not necessarily an easy task since some unsense variants may be difficult to predict from sequences and their experimental identification requires more experiments than customary at the moment.

## LIST OF ABBREVIATIONS

DFEs: Distribution of Fitness Effects
ESEs: Exonic Splice Site Enhancers
ESSs: Exonic Splicing Silencers
GTR: General Time Reversible
GWASs: Genome-wide Association Studies
TF: Transcription Factor
VariO: Variation Ontology
VEP: Variant Effect Predictor
